# Use of a Novel Variable Power Radiofrequency Ablation System Specific for Knee Chondroplasty: Surgical Experience and Two-Year Patient Results

**DOI:** 10.7759/cureus.12864

**Published:** 2021-01-22

**Authors:** Danielle Piper, Clare Taylor, Nick Howells, James Murray, Andrew Porteous, James R Robinson

**Affiliations:** 1 Trauma and Orthopaedics, Avon Orthopaedic Centre, Bristol, GBR; 2 Trauma and Orthopaedics, North Bristol NHS Trust, Bristol, GBR

**Keywords:** chondroplasty, knee arthroscopy, radiofrequency ablation

## Abstract

Introduction

Although stabilisation of knee cartilage lesions (chondroplasty) may be performed with an arthroscopic shaver, more recently, radiofrequency (RF) ablation has gained in popularity. However, their remain some concerns about the avoidance of thermal injury, chondrolysis, and osteonecrosis with the use of RF devices.

Methods

We reviewed the outcomes of 85 knee chondroplasties performed with a new RF ablation wand designed for knee chondroplasty. Lesion details and Chondropaenia Severity Score (CSS) were recorded for each patient. We evaluated the occurrence of adverse outcomes, post-operative complications, and the need for further surgery. Post-operative outcomes scores (Oxford Knee Score [OKS], Knee injury and Osteoarthritis Outcome Score [KOOS], and International Knee Documentation Committee [IKDC] subjective knee outcome) were recorded at a minimum of one-year follow-up.

Results

At the final mean follow-up of 27.5 months (range: 12-46.6 months), 12 (14%) knees had undergone or were listed for further surgery. Four patients had corticosteroid injections for ongoing pain at a median 7.5 months (range: 5-20 months) post-operatively. There were no observed re-operations considered to be caused by complications related to thermal injury. Of the six patients listed for or undergoing knee arthroplasty, five (83%) had grade 4 lesions found at the arthroscopic chondroplasty. A negative correlation was noted between CCS, and post-operative IKDC subjective score (R=-0.35), KOOS Sports (R=-0.39), and KOOS QoL (R=-0.36).

Conclusions

We found that RF chondroplasty appeared safe, and there were no concerns with regard to thermal injury. Functional outcome appeared to be related to the quality of chondral and meniscal tissue throughout all knee compartments, with better results for isolated grade 2 and 3 cartilage lesions.

## Introduction

Focal articular surface lesions in the knee are common [1] and observed in approximately two-thirds of knee arthroscopies [2]. Chondral lesions may result from ageing, disease, and injury [3], often following a deleterious course [4] with progression in size and grade due to mechanical wear [5]. As a known precursor of osteoarthritis, untreated lesions may result in loss of function, pain, and swelling [6-8]. This may eventually lead to the requirement for knee arthroplasty or osteotomy. Given the risk of debilitating outcomes, intervention to stabilise chondral lesions may be warranted in an attempt to slow progression, maintain quality of life, and potentially delay more invasive surgery.

The most common of these interventions is arthroscopic chondroplasty [9], in which loose and fibrillated articular cartilage is removed in an attempt to smooth and stabilise the defect [10,11]. An arthroscopic shaver is often used to mechanically debride the lesion. However, this has been associated with inadvertent removal of adjacent healthy tissue and resultant lesion progression [12,13]. Radiofrequency (RF) ablation is an alternative that has recently gained in popularity for chondroplasty in arthroscopic knee surgery. Activation of the RF electrosurgical wand in a conductive solution (sodium chloride) causes a non-equilibrium plasma vapour layer to form at the tip of the bipolar electrode [14,15]. Current across the vapour layer produces electrons that break down the water molecules into highly energised free radicals, causing disintegration of organic bonds in adjacent tissue and forming gaseous ablation by-products [14]. Studies have suggested that bipolar RF ablation may be advantageous in achieving a smoother surface [16,17], with favourable results in trials comparing its use to that of a shaver [18-20]. However, there have been concerns about thermal injury [21], including chondrocyte death [22] and the development of post-operative chondrolysis and osteonecrosis [23].

Critical in achieving smooth, volumetric tissue removal, with a minimal margin of chondrocyte injury, is the ability to ablate target cartilage tissue with a stable plasma vapor layer around the bipolar electrode [14,24]. Maintenance of a stable plasma involves a complex interaction between current and flow of saline past the tip of the wand. This can be influenced by the wand being connected to the flow pump and control unit in the operating room. Additionally, with older generations of wands, it was necessary to activate the wand away from tissue to avoid thermal injury and then introduce the plasma vapour to target tissue by “hovering” above the cartilage surface in order to ablate it whilst avoiding pressure of the wand against the cartilage surface that could result in thermal injury [22].

In 2014, the Werewolf Generator and Flow 50 plasma wand (Smith and Nephew, Austin, TX, USA) were introduced to our department as part of a limited commercial release to replace an existing RF ablation system already in use. This device was developed with specific utility for knee chondroplasty with ablation modes adapted for specific tissue types (such as cartilage, meniscus, or synovium) and introduced new software to modulate the pump flow for increased plasma stability and ablation control. There are currently limited studies on the safety and functional performance of chondroplasty in large patient series. Novel technologies require studying in the clinical setting to ensure safety and gauge their real-world application. We hypothesised that the use of RF ablation for chondroplasty, when performed alone or in combination with meniscectomy or synovectomy, would be safe without complications such as thermal injury. We also wished to ascertain whether the overall quality of chondral and meniscal tissue throughout all knee compartments was related to post-operative outcome.

## Materials and methods

We identified patients from our research database who had undergone arthroscopic chondroplasty using the Werewolf generator and Flow 50 Coblation wand during a three-year period beginning from July 2014, when the device was introduced. The study was registered with our Local Patient Safety Assurance and Audit Clinical Governance Department (project number CE52507). Inclusion criteria were all patients undergoing knee arthroscopic chondroplasty with or without treatment of a meniscal lesion or synovectomy. Exclusions included associated ligament reconstruction and high tibial osteotomy. Patients with a chondral lesion stabilised using RF chondroplasty and then further treated with an additional cartilage procedure, such as microfracture or autologous matrix-induced chondrogenesis, were included. We used the electronic patient record (Bluespier, Droitwich, Worcestershire, UK) to review the operative notes and clinic letters to retrieve clinical information, the types of pathology treated, and whether any intra-operative complications were reported. Patient demographic data (age, sex, side of surgery), operative data (location, size, and grade of chondral of lesions), meniscal pathology, and treatment were recorded. In addition, during the study period, the operating surgeons were asked to record any intra-operative adverse event.

Surgical technique 

The arthroscopic procedures were undertaken by seven fellowship-trained consultant knee surgeons working in our department. After initial inspection of the joint, meniscal and chondral lesions were addressed. The chondral surfaces were probed and graded using the Outerbridge score [25]. ﻿The Chondropaenia Severity Score (CSS) [26], representing the quality of chondral and meniscal tissue throughout all knee compartments, was calculated for each patient. Chondral lesions with unstable flaps at their periphery were treated by removal of the flaps to create a smooth stable margin at the periphery of the lesion. Areas of chondral fibrillation were smoothed.

**Figure 1:**
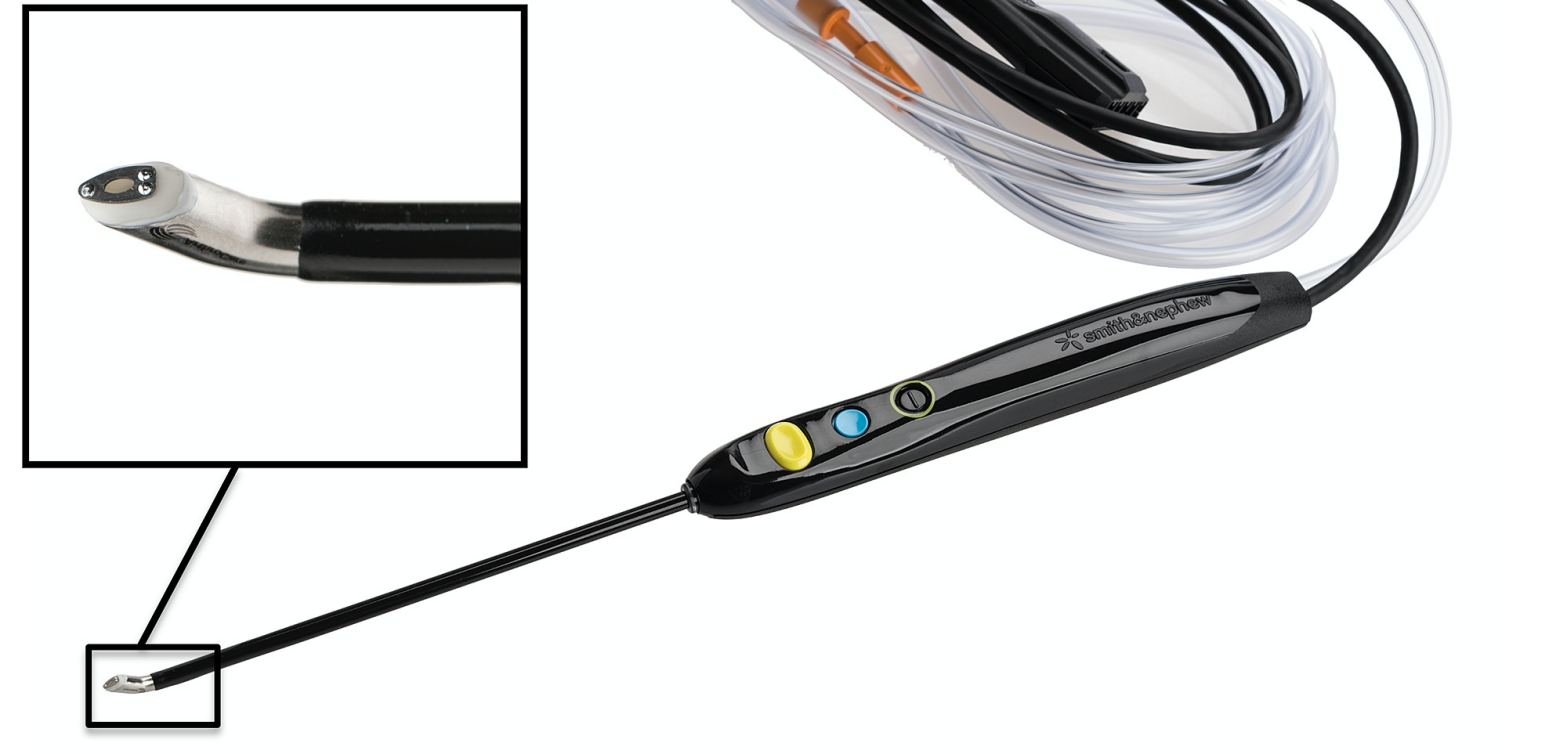
The Flow 50 RF ablation wand. The ceramic bevel at the tip of the wand allows the tip to rest on the joint surface without the electrode contacting the tissue. This design feature is aimed at improving the controlled introduction of target cartilage tissue into the stable plasma vapour layer adjacent to the bipolar electrode. RF, radiofrequency

The Werewolf generator and Flow 50 Coblation wand is designed specifically for use in the knee with variable power and flow settings. The generator has five operating modes that adjust the power and flow at the tip of the wand for use with different tissue types. The “Low” setting is indicated for use on articular cartilage. The wand has a 45-degree ceramic bevel at its tip (Figure 1) that may rest against the articular surface without the electrode contacting it, thus aiding the precision to the depth of tissue removal. The wand was activated and moved continuously with the aim of smoothing the flaps at the periphery of the chondral lesion. Both the intra-articular fluid and outflow temperatures were monitored within the hand piece. The controller was set to emit an alarm if the temperature exceeded ﻿30°C. Once the chondroplasty was performed, the ﻿chondral surface was probed to ensure smooth and stable margins at the periphery of the lesion.

Study outcomes

The primary outcome measures were ﻿the requirement for further surgery and complications related to the use of RF chondroplasty or surgery. The following patient-reported outcome measures (PROMs) were collected for all patients with a minimum 12-month follow-up as secondary outcome measures: Oxford Knee Score (OKS), Knee injury and Osteoarthritis Outcome Score (KOOS), and International Knee Documentation Committee (IKDC) subjective knee outcome. Patients were questioned about further operative interventions following RF chondroplasty and further details obtained from the electronic patient record. All patients were contacted by post with a letter explaining the study and providing PROMs questionnaires with a return, stamped envelope enclosed. Patients who had not returned completed outcome scores within two weeks were contacted by telephone. Three attempts were made to contact the patients by telephone before being documented as lost to follow-up.

Statistical analysis

All scores were entered into an Excel spreadsheet (Microsoft, Redmond, WA, USA), which was used to perform the statistical analysis. Student’s t-test was used to compare differences in post-operative IKDC score between CCS grades. Pearson’s correlation coefficient was used to assess factors associated with patient-reported outcome scores. A p-value of <0.05 was considered statistically significant.

## Results

A total of 82 (28 females, 54 males; mean age: 41 years; range: 16-67 years) patients identified from the database met the inclusion criteria. Three patients underwent bilateral procedures. The details of the 85 operative procedures undertaken are provided in Table 1.

**Table 1:** Operative procedures (n=85) ACL, anterior cruciate ligament

Procedure	N
Isolated chondroplasty	35
Chondroplasty and meniscectomy	25
Chondroplasty and meniscus repair	7
Chondroplasty and removal of loose body	4
Chondroplasty and microfracture/ autologous matrix-induced chondrogenesis	4
Chondroplasty and excision of plica	3
Chondroplasty and ACL graft plasty	3
Chondroplasty, meniscectomy, and removal of loose body	2
Chondroplasty and drilling of sub-chondral cyst	1
Chondroplasty and removal of patella tension band wires	1

Chondral lesions treated

Of the study cohort, 55 (67.1%) patients had one chondral lesion treated, 25 (30.5%) had two chondral lesions treated, and two (2.5%) had three lesions. The mean CSS of the cohort was 83.5 (standard deviation = 9.8; range = 58-97). The breakdown of chondral lesions treated is shown in Table 2.

**Table 2:** Cartilage lesions treated by CSS score CSS, ﻿Chondropaenia Severity Score

	Number of chondral lesions treated	Mean lesion size
CSS grade A (85–100 points), n = 34	1 lesion (82%); 2 lesions (18%)	190 mm^2^
CSS grade B (71–84 points), n = 40	1 lesion (67.5%); 2 lesions (30%); 3 lesions (2.5%)	304 mm^2^
CSS grade C (56–70 points), n = 8	2 lesions (88%); 3 lesions (12%)	237 mm^2^

Further interventions and complications

At the final mean follow-up of 27.5 months (range: 12-46.6 months), 12 knees had undergone or were listed for further surgery: five total knee replacement, one patellofemoral joint replacement, three high tibial osteotomies, and three arthroscopies (Table 3). Four patients had corticosteroid injections for ongoing pain at a median of 7.5 months post-operatively (range: 5-20 months).

**Table 3:** Details of further surgery (n=12) HTO, high tibial osteotomy; PFJ, patellofemoral joint replacement; TKR, total knee replacement

Patient demographics	Details of primary surgery	Further surgery (post-operative month)
A 55-year-old female	Chondroplasty of 240 mm^2^ grade IV medial femoral condyle lesion and medial tibial plateau lesion. Degenerative medial meniscus tear resected	TKR (24 months)
A 55-year-old female	Chondroplasty of 200 mm^2^ grade II medial femoral condyle lesion and 100 mm^2^ grade III lateral tibial plateau lesion	TKR (at another institution, month not available)
A 67-year-old male	Chondroplasty of 400 mm^2^ grade IV trochlear lesion and grade II lateral femoral condyle lesion	TKR (23 months)
A 50-year-old male	Chondroplasty of 150 mm^2^ grade IV medial femoral condyle lesion and 180 mm^2^ grade IV trochlear lesion	Listed for TKR (21 months)
A 47-year-old male	Chondroplasty of 200 mm^2^ grade IV medial femoral condyle lesion and 400 mm^2^ grade IV trochlear lesion	TKR (7 months)
A 45-year-old female	Chondroplasty of 400 mm^2^ grade IV trochlear lesion and 400 mm^2^ grade IV patella lesion	Listed for PFJ replacement (38 months)
A 32-year-old male	Chondroplasty of chondral flaps around previous osteochondral mosaicplasty of the medial femoral condyle	Listed for HTO (46 months)
A 23-year-old female	Chondroplasty of 100 mm^2^ grade IV chondral lesion and lateral femoral condyle lesion	Medial closing wedge HTO (9 months)
A 30-year-old male	Chondroplasty of 420 mm^2^ grade IV medial femoral condyle chondral lesion	Medial opening wedge HTO (12 months)
A 23-year-old male	Chondroplasty of 25 mm^2^ medial femoral condyle chondral lesion and medial meniscus repair	Arthroscopy for further medial meniscal tear (10 months)
A 32-year-old male	Chondroplasty of 400 mm^2^ osteochondritis dissecans lesion in the medial femoral condyle	Listed for further arthroscopic chondral surgery
A 54-year-old male	Chondroplasty of 100 mm^2^ grade II patella chondral lesion	Arthroscopic washout of haematoma (2 weeks)

There were no observed re-operations considered to be caused by complications related to the study device. One intra-operative complication was noted: the tip of the electrode dissociated from the wand. This was immediately identified, the tip was retrieved, and the wand and broken electrode were returned to the manufacturer for investigation. No obvious defects were found; it was presumed that the tip of the electrode might have been damaged when introduced the knee. No other safety concerns were reported by the operating surgeons. Additionally, in the subsequent four total knee arthroplasties performed at our institution, there were no findings suggestive of chondrolysis or osteonecrosis.

PROMs

At the final follow-up, the following PROMs were obtained: 42 replies for the OKS and 44 replies for the KOOS and IKDC (Table 4). Two patients failed to complete the OKS fully and did not respond to further attempts to provide complete scores. Also, 37 (32%) patients did not answer the three follow-up calls or hung up, seven (8%) did not have their scores taken because they had undergone additional surgery, and four (5%) declined to complete PROMs scores but reported that their knee was functioning well.

**Table 4:** Post-operative PROMs data ADL, activities of daily living; IKDC, International Knee Documentation Committee; KOOS, Knee injury and Osteoarthritis Outcome Score; OKS, Oxford Knee Score; PROM, patient-reported outcome measure

	N	Mean	Range	Median	Standard deviation
OKS (0=worst; 48=best)	42	35	7–48	38	11
KOOS Symptoms (0=worst; 100=best)	44	64	0–100	64	26
KOOS Pain (0=worst; 100=best)	44	68	3–100	69	24
KOOS ADL (0=worst; 100=best)	44	77	4–100	82	23
KOOS Sports (0=worst; 100=best)	44	52	0–100	53	31
KOOS QoL (0=worst; 100=best)	44	45	0–100	44	29
KOOS Total (0=worst; 100=best)	44	61	5–100	61	24
IKDC (0=worst; 100=best)	44	55	8–98	56	24

A statistically significant negative correlation was noted between CCS and post-operative IKDC subjective score (R=-0.35), KOOS Sports (R=-0.39), and KOOS QoL (R=-0.36) (Table 5). No statistically significant correlation was found between CCS score and the other KOOS domains or the OKS. No statistically significant correlation was found between lesion size, number, age, and outcome score. There were no significant differences in post-operative PROM scores between patients undergoing isolated chondroplasty and those undergoing combined chondroplasty and meniscectomy.

**Table 5:** Median PROMs scores by Chondropaenia Severity Score ADL, activities of daily living; CSS, ﻿Chondropaenia Severity Score; IKDC, International Knee Documentation Committee; KOOS, Knee injury and Osteoarthritis Outcome Score; QoL, quality of life; OKS, Oxford Knee Score; PROM, patient-reported outcome measure

	CSS grade		
PROMs	A	B	C
OKS (0=worst; 48=best)	42 (range 20–48; SD = 9)	35 (range 7–47; SD = 10)	28 (range 7–44; SD = 11)
KOOS Symptoms (0=worst; 100=best)	80 (range 30–100; SD = 24)	65 (range 0–100; SD = 25)	50 (range 0–75; SD = 21)
KOOS Pain (0=worst; 100=best)	86 (range 28–100; SD = 20)	68 (range 3–100; SD = 24)	61 (range 20–48; SD = 9)
KOOS ADL (0=worst; 100=best)	94 (range 29–100; SD = 17)	78 (range 4–100; SD = 25)	71 (range 20–48; SD = 9)
KOOS Sports (0=worst; 100=best)	75 (range 35–100; SD = 27)	45 (range 0–85; SD = 30	35 (range 0–48; SD = 9)
KOOS QoL (0=worst, 100=best)	56 (range 31–100; SD = 26)	28 (range 0–81; SD = 27)	19 (range 0–62; SD = 25)
IKDC (0=worst; 100=best)	78 (range 51–99; SD = 16)	55 (range 8–84; SD = 19)	42 (range 8–70; SD = 18)

## Discussion

There are limited studies in the literature on RF ablation chondroplasty in the knee. The U.K. National Institute of Health and Care Excellence (NICE) 2014 guidance [21] recommended that there appeared to be short-term benefits, although clinicians should pay particular attention to the avoidance of thermal injury. It was stated that there was a need for further research and in particular that there was insufficient evidence for the use of arthroscopic RF chondroplasty in older patients with osteoarthritis. Our study reinforces these recommendations and found that chondroplasty using the Flow 50 plasma ablation wand and Werewolf generator, particularly for patients with higher CSS (indicating better quality of chondral and meniscal tissue throughout all knee compartments), appeared safe for the treatment of small, isolated chondral defects.

RF ablation as an alternative to mechanical debridement has recently gained in popularity for chondroplasty in arthroscopic knee surgery. Studies have suggested that, compared with the use of a mechanical shaver, a macroscopically smoother surface may be obtained [16,17,22,27] with reduced cartilage permeability and maintenance of cartilage structural properties [28]. In a randomised controlled clinical study comparing the use of RF ablation to a mechanical shaver for chondroplasty at the time of meniscectomy [18-20], PROMs were improved and there were fewer re-operations. Voloshin et al. [29] undertook second-look arthroscopies two years following RF ablation chondroplasty and found that the majority (88%) of lesions showed no sign of progression and 56% appeared improved with complete or partial filling. Although there have been concerns about thermal injury to cartilage with bipolar RF [17,22,23], Amiel et al. [24] found that with optimal wand usage, thermal penetration in the treatment area may be minimised, limiting chondrocyte death to approximately 125 µm compared with approximately 250 µm with a mechanical shaver.

Maintaining a stable plasma vapour layer was challenging with older RF wand generations. Operating room suction units can cause too much suction at the tip of the bipolar electrode to allow stable plasma formation, and the tip of the wands has to be held “hovering” above the cartilage surface in order to introduce the plasma vapour layer yet avoid pressure of the wand against the cartilage and subsequent thermal injury. Despite this, chondroplasty series with older wand generations have shown acceptable results [10,11,15,19].

It is important to monitor all new surgical techniques and devices as they are introduced into clinical practice, particularly due to the concerns regarding the risk of thermal damage to surrounding cartilage when using RF within the knee. Our study demonstrated device safety with PROMs results at a mean follow-up of 27.5 months, which is comparable to other case series of RF chondroplasty with much shorter follow-up (three months) [15]. None of the surgeons reported safety concerns, and we found no evidence of concerns with thermal injury that have been reported in other series [23].

There were a number of re-operations in our cohort of patients. Of the patients who went onto joint arthroplasty, five out of the six had large grade 4 chondral lesions found at surgery. In these cases, arthroscopic assessment of the knee was undertaken where pre-operative imaging had failed to demonstrate full-thickness chondral lesions or to determine suitability for uni-compartmental arthroplasty. In some cases, there had been progression of the cartilage lesion whilst the patient had been on a surgical waiting list. In these cases, which were found to have lower CSS, the outcomes were poor. RF chondroplasty is not indicated for grade 4 disease when this is part of established osteoarthritis and the minimal efficacy of arthroscopic procedures, where there is advanced degenerative change, is increasingly understood [30]. Although previous comparative studies of the outcomes of RF chondroplasty have excluded patients with grade 4 lesions [10,11], our series suggests that for small, isolated grade 4 chondral lesions, RF chondroplasty may be reasonable in order to stabilise them and to ascertain suitability for further biological treatment or focal resurfacing.

This study is limited by its retrospective design and the lack of pre-operative PROM scores. Additionally, we did not undertake a comparison between different types of RF wand. However, our study does allow comparison of post-operative outcomes with previously published chondroplasty cohorts. ﻿Gharaibeh et al. [15] retrospectively analysed a cohort of patients undergoing chondroplasty with an older generation of RF ablation device and reported a statistically significant improvement in normalised total KOOS from a mean pre-operative score of 45.9 (SD: 16.8) to mean post-operative score of 65.6 (SD: 18.9) at an average follow-up of 4.2 months post-operatively. The mean post-operative total KOOS score of 61.3 (SD: 24) we found in our study, at much longer-term follow-up (mean: 27.5 months), compares reasonably well, particularly as KOOS scores following chondroplasty are known to diminish from one-year post-operative [18]. Unlike Gharaibeh et al. we did not find poorer outcomes in those patients undergoing combined meniscectomy and chondroplasty, although we found a lower CSS score was associated with poorer post-operative PROMs.

## Conclusions

The Werewolf/Flow 50 system for RF knee chondroplasty appears safe within routine clinical practice, with no specific concerns with regard to thermal injury. Functional post-operative outcomes relate to the quality of chondral and meniscal tissue throughout all knee compartments. Correlation between higher CSS at surgery and better post-operative function (IKDC subjective score and KOOS sports and quality-of-life domains) further supports the recommendation that RF chondroplasty is better suited for the stabilisation of discreet chondral lesions in knees with minimal deterioration in other compartments. There is decreased utility in patients with osteoarthritis, particularly if widespread. Further work is needed to assess the long-term outcomes of RF chondroplasty with this system.
